# Screening and Characterization of RAPD Markers in Viscerotropic *Leishmania* Parasites

**DOI:** 10.1371/journal.pone.0109773

**Published:** 2014-10-14

**Authors:** Imen Mkada–Driss, Ramzi Lahmadi, Ahmed S. Chakroun, Chiraz Talbi, Souheila Guerbouj, Mehdi Driss, Elwaleed M. Elamine, Elisa Cupolillo, Moawia M. Mukhtar, Ikram Guizani

**Affiliations:** 1 Laboratoire d'Epidémiologie Moléculaire et de Pathologie Expérimentale (LR11IPT04)/Laboratoire d'Epidémiologie et d'Ecologie Parasitaire (LR00SP04) –Institut Pasteur de Tunis, Université Tunis el Manar, Tunis, Tunisia; 2 Institute of Endemic Diseases, University of Khartoum, Khartoum, Sudan; 3 Laboratorio de Pesquisas em Leishmaniose, Instituto Oswaldo Cruz, Rio de Janeiro, Brazil; University of Brighton, United Kingdom

## Abstract

Visceral leishmaniasis (VL) is mainly due to the *Leishmania donovani* complex. VL is endemic in many countries worldwide including East Africa and the Mediterranean region where the epidemiology is complex. Taxonomy of these pathogens is under controversy but there is a correlation between their genetic diversity and geographical origin. With steady increase in genome knowledge, RAPD is still a useful approach to identify and characterize novel DNA markers. Our aim was to identify and characterize polymorphic DNA markers in VL *Leishmania* parasites in diverse geographic regions using RAPD in order to constitute a pool of PCR targets having the potential to differentiate among the VL parasites. 100 different oligonucleotide decamers having arbitrary DNA sequences were screened for reproducible amplification and a selection of 28 was used to amplify DNA from 12 *L. donovani*, *L. archibaldi* and *L. infantum* strains having diverse origins. A total of 155 bands were amplified of which 60.65% appeared polymorphic. 7 out of 28 primers provided monomorphic patterns. Phenetic analysis allowed clustering the parasites according to their geographical origin. Differentially amplified bands were selected, among them 22 RAPD products were successfully cloned and sequenced. Bioinformatic analysis allowed mapping of the markers and sequences and priming sites analysis. This study was complemented with Southern-blot to confirm assignment of markers to the kDNA. The bioinformatic analysis identified 16 nuclear and 3 minicircle markers. Analysis of these markers highlighted polymorphisms at RAPD priming sites with mainly 5′ end transversions, and presence of inter– and intra– taxonomic complex sequence and microsatellites variations; a bias in transitions over transversions and indels between the different sequences compared is observed, which is however less marked between *L. infantum* and *L. donovani*. The study delivers a pool of well-documented polymorphic DNA markers, to develop molecular diagnostics assays to characterize and differentiate VL causing agents.

## Introduction

Visceral leishmaniasis (VL) is a vector borne parasitic disease that constitutes a major public health problem in many parts of the world; it is fatal if left untreated or not treated on time. VL is endemic in more than 60 countries with 90% of the 200,000–400,000 annual cases reported in 6 countries in Africa (Ethiopia and Sudan), Indian subcontinent (Bangladesh, India and Nepal) and Latin America (Brazil) [1]. Visceral leishmaniasis is also complicated following treatment by another form of disease, Post Kala Azar Dermal Leishmaniasis (PKDL) in India (10–20%) and East Africa (Sudan (30–50%)) with severe forms being more difficult to treat than VL [2]. Different etiological agents are known to be responsible for VL, belonging to the *Leishmania donovani* complex: *L. donovani*, *L. infantum, L. chagasi (syn. L. infantum)*, and *L. archibaldi* [3]. In East Africa and India, VL has essentially an anthroponotic transmission; in other areas transmission involves (non human) mammalian reservoirs, mostly canids [4]. However in Sudan, different animals were found infected with VL pathogens [5], [6], [7]. While all these parasites cause VL, they have been also identified as causal agents of forms of cutaneous leishmaniasis in different parts of the world [8]–[15]. The transmission cycles of these cutaneous disease forms largely remain poorly elucidated.

Taxonomy of these parasites is a matter of controversy. *L. infantum* is considered as forming its own complex [16] or as being one of the only 2 species constituting the *L. donovani* complex [17]. DNA studies question its presence in Sudan (and East Africa) and hypothesize converged evolution of isoenzymes to explain the zymodeme classification of these parasites [18]. It is also agreed that *L. chagasi* is synonymous to *L. infantum* [19]. Ranking of *L. archibaldi* as a species is questioned by DNA analyses that propose to consider it as an *L. donovani* species [17], [18], [20], [21]. Nevertheless these parasites belong to a zymodeme that is exclusive to, and well present in Sudan and Ethiopia [12], [22], [23] and thus may contribute there to the higher prevalence and earlier observation of PKDL cases than in the Indian subcontinent.

Although in general at a country/region level, VL is associated to only one species, in some areas (e.g. East Africa, Middle East, China) these pathogens may co-exist [1], [23]. These species are difficult to differentiate in a single step [24], [25] and their differentiation is generally based on numerical taxonomy or population genetics using multiple markers (eg microsatellites; multilocus sequence analysis; random markers, etc) [18], [20], [21], [26], [27], [28]. Therefore, identification of DNA markers that could differentiate among the VL causing agents according to their taxonomy or eco-epidemiological or clinical features is highly relevant to disease control, epidemiological investigations and travel medicine.

Random Amplification of Polymorphic DNA (RAPD) is a simple and rapid technique that does not require prior knowledge on the genomes to characterize organisms, using one randomly determined (usually a decamer) primer [29], [30]. It is used for genetic characterization of a range of organisms, plants, animals or microorganisms for diverse purposes [31]–[35], In case of *Leishmania*, taxonomy and typing were major applications [26], [36]–[38]. In the Old World, different levels of inter– or intra– specific discrimination of *Leishmania* parasites could be observed [38], which were supposed to be due to clustered organization of different kinds of repetitive motives [39]. Priming sites for amplification with the 10–mers could be either polymorphic among parasite isolates or unique to some of them, therefore leading to a differential amplification resulting in different levels of discrimination [38], [39]. It has been used alone or with other tools to investigate intra–taxonomic (complex, species, zymodeme) diversity of VL parasites [26], [40]–[42], which highlighted their diversity and their genetic differentiation according to their geographical origin. It proved also an effective method to obtain genetic markers to develop relevant *Leishmania* DNA assays [39], [43], [44]. Therefore, this technique appeared as an adequate tool for comparison of VL parasites from diverse geographical settings and the selection of DNA markers among differentially amplified bands, which may be relevant for the development of DNA assays for the differentiation of/among VL parasites. This approach was all the more justified in absence of thorough genome information as regards such parasites.

This study reports on the selection of 28 RAPD primers for the amplification of a panel of DNA from geographically diverse VL parasites and the characterization of 22 cloned and sequenced RAPD markers using the existing genome resources. The sequences were first compared to their hits on the available genomes of *L. infantum* and *L. donovani*, and then to the ones on *L. major* to annotate the markers as regards to their chromosomal assignment, genomic context, and content. In some cases, Southern blot analysis confirmed assignment of the DNA markers to *Leishmania* DNA, precisely to kDNA. The analysis confirmed occurrence of polymorphisms at the priming sites and within the markers and allows predicting potential of these markers as targets for DNA assays aiming at the characterization of viscerotropic *Leishmania* species.

## Materials and Methods

### Ethics statement

Parasite strains used in this study are from different geographical origins. Parasites from Tunisia (LV10 and Drep14, Table 1) were isolated for the diagnosis of patients having VL or CL in Tunisian hospitals [45], [46]. Strains from Sudan (MW9, MW26, MW106 and MW3, Table 1) belong to human parasite collections that were isolated in medical centers at diagnosis of VL, CL or PKDL patients during a previous study supported by TDR program. All these strains obtained to confirm the diagnosis of leishmaniasis patients in clinical settings were cryopreserved under anonymous codes. Parasite strains GEBRE1, JEDDAH-KA, H9, DEVI, LRC-L57 and ADDIS164 (Table 1) were kindly provided from the *Leishmania* reference center in Montpellier, France (Pr. Jean Pierre Dedet). This study who received the support of TDR program also received the approval of the ethical committees of the Institut Pasteur de Tunis, of the Ministry of Health in Sudan and the WHO (Geneva).

**Table 1:** Panel of *Leishmania* strains used for screening of RAPD markers.

WHO code	Codes assigned in other studies	Zymodeme	Pathology	Species
MHOM/SD/00/MW9		MON–82	VL	*L. archibaldi*
MHOM/SD/00/MW26		MON–82	PKDL	*L. archibaldi*
MHOM/ET/72/GEBRE1	ARC-11/LEM1005/D28/ARC-43 (LG11)	MON–82	VL	*L. archibaldi*
MHOM/SD/00/MW106		ND	VL	*-*
MHOM/SD/00/MW3		ND	CL	*-*
MHOM/TN/93/LV10		MON–80	VL	*L. infantum*
MHOM/TN/97/Drep14		MON–24	CL	*L. infantum*
MHOM/SA/81/JEDDAH-KA	DON-31/LEM536/D32/DON81	MON–31	VL	*L. donovani*
MHOM/KE/75/H9	LEM496/D31	MON–32	VL	*L. donovani*
MHOM/IN/00/DEVI	DON-09/LEM138/DON39/LG9	MON–2	VL	*L. donovani*
IMRT/KE/62/LRC-L57	LEM719/D21	MON–37	VL	*L. donovani*
MHOM/ET/84/ADDIS164	LEM980/D29	MON–83	VL	*L. donovani*

WHO that summarizes Host, geographical origin, year of isolation and laboratory code is presented together with pathology and zymodeme code whenever available. MON– corresponds to zymodeme code attributed by the reference center in Montpellier. The table also gathers study codes assigned to some of the isolates in other studies: D21, D28, D29, D31 and D32: strains used in [20]; DON-39 and ARC-43: strains used in [21]; Devi, H9, LRC-L57, ADDIS 164: strains used in [18]; Devi, GEBRE1 and KA-Jeddah: strains used in [53]; DON-81and ARC-43 (LG11): strains used in [28]; DON-09, DON-31, DON-39 and ARC-11: strains used in [25]. Country abbreviations are shown as specified by WHO recommendations (SD: Sudan; TN: Tunisia; ET: Ethiopia; SA: Saudi Arabia; KE: Kenya; IN: India). ND: Not Determined; CL: cutaneous leishmaniasis; VL: visceral leishmaniasis; PKDL: Post Kala azar Dermal Leishmaniasis.

### Parasites and DNA

Twelve *Leishmania* strains having different geographical origins belonging to *L. infantum*, *L. donovani* or *L. archibaldi* species according to their zymodeme were selected for the purpose of the study (Table 1). Among these, 5 *L. donovani* originating from Kenya, Ethiopia, Saudi Arabia and India and one *L. archibaldi* originating from Ethiopia were kindly provided by the *Leishmania* reference center of Montpellier (Pr. Jean Pierre Dedet); the others were obtained from human parasite collections in Sudan (N = 4) with two typed as *L. archibaldi* (MON-82) in Rome, or Tunisia (N = 2) that were typed at the Institut Pasteur d'Algérie (Dr. Zoubir Harrat) as *L. infantum* MON-24 and MON-80. Two isolates selected from field collections in Sudan, MW106 and MW3, were not typed by isoenzyme analyses but were assigned to *L. infantum*/*L. donovani* complex by DNA techniques. The parasites were cultured in RPMI 1640 supplemented with 15% fetal calf serum, 2 mM glutamine, 100 U/ml penicillin/50 U/ml streptomycine, at 22°C. The total DNAs were purified using phenol–chloroform extraction and ethanol precipitation [47]. Parasite species identification was performed on these DNA preparations using at least one DNA technique previously described [24], [27], [47].

### RAPD primers and amplification

One hundred 10–mer primers corresponding to five randomly selected kits defined by Operon technologies (Qiagen, GmbH, Germany) (OP-AD, OP-AY, OP-U, OP-E, and OP-O) were commercially synthesized and used as primers in RAPD experiments. All primers used were resuspended in TE buffer, stored at −20°C, and 10 µM (10 pmol/µl) working solutions were prepared. These primers were screened over two independent amplification rounds on one or two *L. archibaldi* DNAs and 28 primers giving reproducible profiles were selected (Table S1). Subsequently stability of the profiles was inferred upon this screening step. The same person did the RAPD screening and amplification using a single protocol. The RAPD amplification protocol was as described previously [38]. In brief, RAPD amplifications were processed in 50 µl reactions containing 0.1 mM of each dNTP, 0.8 µM primer, 2.5 U *Taq* DNA polymerase (Promega–Biogene, Tunis, Tunisia), 1X buffer supplied with the enzyme, containing 1.5 mM MgCl_2_, and 20 ng genomic DNA. Amplification was processed in a MJ research PTC-150 Minicycler (MJ research, Inc., USA). The amplification started with a denaturation at 94°C for 5 minutes, then 45 cycles were run at 94°C for 1 minute, 36°C for 1 minute and 72°C for 2 minutes; and a final elongation was performed at 72°C for 8 minutes. The RAPD products were separated by 1.6% agarose gel electrophoresis and visualized under UV light after staining with ethidium bromide. Interpretation of the patterns was based on the presence or absence of amplified DNA bands. Different readers analyzed the gels and we used a conservative reading of the gels taking account of intense and faint bands.

### Phenetic analyses of the RAPD results

The bands observed in the gels were scored as absent (0) or present (1); the analysis included all bands regardless of their relative intensity. As RAPD products are considered as dominant markers, similarity indexes according to Nei and Li (NLi) or Jaccard (Ji) were estimated using the MVSP software package (Multi Variate Statistical Package 3.1) [48], thus taking into account only the present bands. The distance matrix was used to establish the phenetic relationship and draw dendrograms using the unweighted pair-group method with arithmetic means (UPGMA), also using the MVSP package.

### Cloning and sequencing of RAPD bands

A selection of 65 differentially amplified bands among the 12 DNAs panel were cut from agarose gels, and purified using the Qiaquick Gel extraction kit (Qiagen, Hilden, Germany) according to the protocol provided by the supplier. In some instances, the bands were re-amplified using the corresponding RAPD primers before the purification step. The bands were cloned into the plasmid vector provided in the pMOSBlue Blunt Ended Cloning Kit (HVD-Amersham, Athens, Greece) following the manufacturer instructions. PCR colony using plasmid primers and agarose gel electrophoresis allowed screening for inserts having the same size than those aimed for the cloning. Only 22 bands generated with 9 RAPD markers were successfully cloned. Analysis of recombinant plasmids was performed by extracting plasmid DNA from small volume cultures grown overnight at 37°C by using the Qiaprep Spin plasmid kit (Qiagen, Hilden, Germany). Sequencing of RAPD markers was performed using the BigDye Terminator v3.1 Cycle Sequencing Kit (HVD-Amersham, Athens, Greece) using the T7 (5′ CTAATACGACTCACTATAGGG 3′) and U19 (5′ TTTTCCCAGTCACGACGT 3′) primers located on the plasmid. The automated sequencer ABI PRISM 3130 Genetic 177 Analyzer (Applied Biosystem, Foster City, CA, USA) was used to generate the electrophoregrams.

### Southern blotting of genomic RFLP and PCR products

Southern blot analyses were performed by digesting 8 µg of DNA of 5 parasite strains representative of the *L. major* (MHOM/TN/90/FMH), *L. archibaldi* (MHOM/ET/72/GEBREI), *L. donovani* (MHOM/ET/67/HU3), *L. tropica* (MCAN/IN/71/DBKM), and *L. infantum* (MHOM/FR/78/LEM1163) species with 50–100 units *EcoR*I, *Pst*I, *Hind*III or *Xho*I (HVD-Amersham, Athens, Greece) restriction enzymes, as previously described [42]. Fragments were separated on 1% agarose gel overnight and transferred to Hybond N+ nylon membrane (HVD, Athens, Greece) as previously described [47]. Some inserts were sequentially used as probes upon labeling with α^32^P dCTP (HVD-Amersham, Athens, Greece) using the Megaprime DNA labeling kit (HVD-Amersham, Greece). Hybridization and dehybridization were done as previously described [47]. Hybridization patterns were obtained upon exposure of the blots to autoradioagraphic films (Kodack XAR5 and XLS films, HVD Amersham, Greece). Absence of signals on the blots upon dehybridization was also confirmed by autoradiography. In order to confirm the minicircle origin of the 3 markers M106/200/OAPY9, LEM536/320/OPAY8 and LEM496/300/OPAD17, total DNAs extracted from 5 strains representative of the species *L. major*, *L. infantum* and *L. donovani* were amplified in 3 identical reaction conditions with the Kinetoplast specific primers AJS81 (5′ GGGGTTGGTGTAAAATAGGGCCGG 3′) and DBY (5′ CCAGTTTCCCGCCCCGGAG 3′) as previously described [10], [11]. PCR products of each set of reaction were separated on different 1.4% agarose gels and the 3 gels were transferred to Hybond N+ Nylon membrane. The 3 cloned markers were individually labeled with α^32^P dCTP and used to hybridize one of the 3 membranes. Results were analyzed by autoradiography and hybridization profiles were compared to the results on agarose gels.

### Bioinformatic analyses

Plasmid sequences were manually removed from each insert sequence generated. These sequences were then submitted to BLAST analysis for homology searches on a local bioinformatics toolbox [49] constituted by updated versions of the different *L. major*, *L. infantum*, and *L. donovani* genomes downloaded from the Sanger site (http://www.sanger.ac.uk/) and a set of bioinformatics programs. We used the Artemis [50], ACT [51] and ClustalW [52] software for annotation, visualization of multilevel comparison of the sequences to the genomic hits, and multiple sequence alignment, respectively. Sequences were also compared to their corresponding hits on the genomes as regards point mutations or indels using a homemade script (Mutation checkup); presence of microsatellites was screened using a script (Misa cluster) developed based on Misa program [53]. Simple sequence repeats were considered as microsatellites when the number of repeats was ≥10 for mononucleotide motives, ≥6 for dinucleotide motives, ≥4 for trinucleotide motives, and ≥3 for tetra and pentanucleotide motives [54]. Imperfect microsatellites correspond to imperfect repeat sequences (eg: (T)6(TG)6C(TG)7*). The sequences of the markers were submitted to GeneBank under the accession numbers reported in Table 2.

**Table 2:** Homology analysis of the sequences of the cloned RAPD markers in 3 *Leishmania* genomes.

		Location	Query coverage	Identity percent	Marker mapping on *L. infantum JPCM5*	Accession number
	Markers	*L. donovani*	*L. infantum*	*L. major*	*L. donovani*	*L. infantum*	*L. major*	*L. donovani*	*L. infantum*	*L. major*	Gene	Gene hit	Gene	
		BPK282A1	JPCM5	Friedlin	BPK282A1	JPCM5	Friedlin	BPK282A1	JPCM5	Friedlin	upstream		downstream	
*L. archibaldi*	L1005/220/OPAY8	LdBK30	LinJ30	LmjF30	99%	99%	98%	222/225 (99%)	218/226 (96%)	200/234 (85%)	LinJ.30.2570		L.inJ.30.2560	KJ525724
											LinJ.30.1470		LinJ.30.1480	
	L1005/320/OPAY8	LdBK30	LinJ30	LmjF30	99%	99%	99%	257/261 (98%)	257/261 (98%)	242/262 (92%)	LinJ.30.1480		LinJ.30.1419	KJ525725
											LinJ.30.1419		LinJ.30.1500	
	L1005/650/OPAY14	LdBK25	LinJ25	LmjF25	98%	98%	93%	609/621 (98%)	613/621 (99%)	546/597 (91%)		LinJ.25.0630		KJ525726
	L1005/1000/OPU10	LdBK05	LinJ05	LmjF05	99%	99%	93%	949/980 (97%)	953/978 (97%)	441/500 (88%)	LinJ.05.0660		LinJ.05.0670	KJ525727
	M106/950/OPAD17	LdBK36	LinJ36	LmjF36	92%	92%	51%	469/481 (98%)	470/482 (98%)	412/514 (80%)	LinJ. 36.4750		LinJ.36.4760	KJ525728
	M106/200/OAPY9	NF	NF	NF	NF	NF	NF	NF	NF	NF	NF	NF	NF	KJ525729
*L. donovani*	LEM138/400/OPAY5	LdBK20	LinJ20	LmjF20	98%	98%	98%	380/391 (97%)	379/391 (97%)	345/395 (87%)	LinJ.20.1300		LinJ.20.1310	KJ525730
	LEM138/550/OPAY14	LdBK35	LinJ35	LmjF35	99%	95%	84%	550/552 (99%)	240/240 (100%)	484/556 (87%)	LinJ.35.1470		LinJ.35.1480	KJ525731
	LEM536/320/OPAY8	NF	NF	NF	NF	NF	NF	NF	NF	NF	NF	NF	NF	KJ525732
	LEM980/320/OPAY8	LdBK36	LinJ36	LmjF36	97%	97%	98%	283/283 (100%)	280/285 (98%)	236/286 (83%)	LinJ.36.5800		LinJ.36.5790	KJ525733
	LEM496/300/OPAD17	NF	NF	NF	NF	NF	NF	NF	NF	NF	NF	NF	NF	KJ525734
	LEM719/1000/OPU3	LdBK23	LinJ23	LmjF23	99%	99%	98%	956/970 (99%)	956/970 (99%)	837/970 (86%)	LinJ.23.1060		LinJ.23.1050	KJ525735
*L. infantum*	LV10/500/OPAD1	LdBK20	LinJ20	LmjF20	98%	98%	80%	436/444 (98%)	435/444 (98%)	330/361 (91%)		LinJ.20.1430		KJ525736
	LV10/700/OPU10	LdBK32	LinJ32	LmjF32	98%	98%	97%	602/608 (99%)	604/608 (99%)	547/605 (90%)	LinJ.32.0870		LinJ.32.0880	KJ525737
	LV10/750/OPAY14	LdBK17	LinJ17	LmjF17	99%	99%	99%	697/716 (97%)	705/715 (99%)	590/714 (83%)	LinJ.17.1550		LinJ.17.1560	KJ525738
	D14/800/OPAD17	LdBK32	LinJ32	LmjF32	99%	99%	99%	756/768 (98%)	759/766 (99%)	692/785 (88%)	LinJ.32.3230		LinJ.32.3240	KJ525739
	D14/800/OPE2	LdBK32	LinJ32	LmjF32	99%	99%	99%	756/768 (98%)	759/766 (99%)	692/785 (88%)		LinJ.32.1050		KJ525740
	D14/800/OPU10	LdBK16	LinJ16	LmjF16	99%	99%	99%	736/743 (99%)	740/743 (99%)	720/743 (97%)		LinJ.16.0590		KJ525741
	D14/1300/OPAY8	LdBK29	LinJ29	LmjF29	99%	99%	99%	698/711 (98%)	699/711 (98%)	665/711 (94%)		LinJ.29.1110		KJ525742

NF: Not found. BPK282A1, JPCM5 and Friedlin are names of *L. donovani*, *L. infantum* and *L. major* strains, respectively used for genome sequencing and available in GeneDB.

## Results

### RAPD analysis of a panel of 12 *Leishmania donovani/Leishmania infantum* complex parasites highlights their clustering according to geographical region

**Figure 1:**
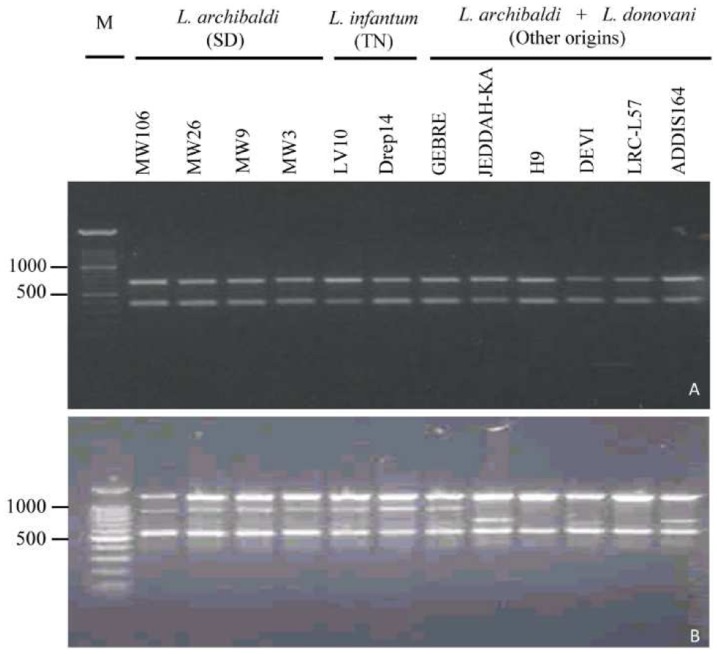
RAPD profiles obtained with primers OP-O7 (A) and OP-O13 (B) to illustrate examples of monomorphic and polymorphic profiles. Laboratory codes of the strains are indicated for each lane. SD: Sudan; TN: Tunisia; M: 100 bp DNA size marker. Indicated sizes are in bp.

A selection of 12 DNAs corresponding to *Leishmania* strains originating from East Africa (Sudan, Ethiopia, and Kenya), India and North Africa (Tunisia) were analyzed by RAPD using 28 decamer primers. According to the primer, the number of amplified bands varied from 1 to 11. The patterns obtained included bands having high intensity and others that were faint or less intense. Only 7 primers provided monomorphic profiles. Twenty–one primers generated RAPD products that allowed different levels of taxonomic differentiation. Figure 1 illustrates examples of such profiles. With OP-O7 primer, the RAPD generated monomorphic products (Figure 1A) while OP-O13 primer generated 3 polymorphic profiles; a first one shared by *L. archibaldi* and *L. infantum* parasites and the 2 others corresponding to *L. donovani* strains (Figure 1B).

**Figure 2:**
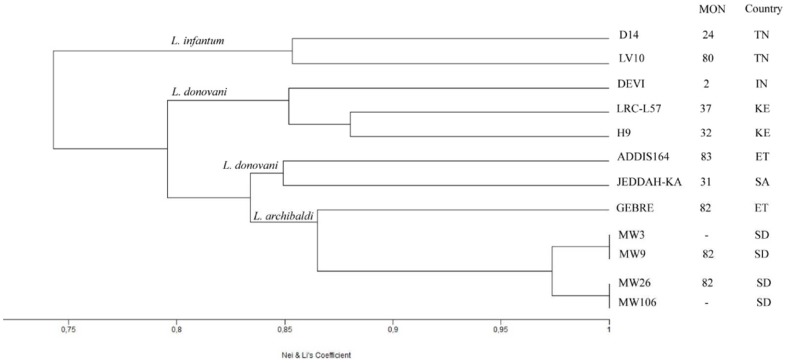
UPGMA dendrogram obtained using Nei and Li similarity indexes of the panel of geographically diverse strains using the RAPD profiles generated with a selection of 9 decamers. Species assignment according to isoenzyme analysis, zymodeme (MON for Montpellier) when available, and country of origin are indicated. ET: Ethiopia; SA: Saudi Arabia; IN: India; KE: Kenya; SD: Sudan; TN: Tunisia.

A total of 155 bands were amplified of which 94 appeared polymorphic (60.65%). Nei and Li similarity indexes (NLi) were estimated and the UPGMA tree obtained highlighted the phenetic relationships between parasites that were clustered according to assignment to species and/or to geographical origins (Figure S1). The range of similarity values (0.772–0.991) indicated very close relationships. However, *L. infantum* and *L. archibaldi* (with the untyped Sudanese parasites) constituted 2 different clusters that grouped together. The Sudanese parasites were differentiated from the Ethiopian strain in the *L. archibaldi* cluster. On this tree, a second group of parasites comprised 2 clusters corresponding to *L. donovani* parasites from Ethiopia and Saudi Arabia on one hand, and Kenya and India on the other (Figure S1). The highest similarity (NLi>0.9) was observed among the Sudanese strains and among the 3 East African *L. donovani* strains, and the lowest ones (0.77<NLi<0.8) were between the *L. infantum* and *L. donovani* strains or between some *L. archibaldi* and the Indian *L. donovani* strains (Table S2). This clustering was conserved with the Jaccard's coefficient (data not shown). When we reduced the dataset to the profiles obtained with the 9 primers that amplified the 22 cloned RAPD bands (here reported), the dendrogram still highlighted a similar clustering of the parasites according to their geographical origin but the relationship of the clusters into taxa was different; similarity values were still high (Figure 2). The L. infantum parasites constituted a group on their own. The L. archibaldi and Sudanese parasites formed a cluster that grouped together with the two clusters of L. donovani parasites, one comprising the strains from Saudi Arabia and Ethiopia, and the other one those from Kenya or India. However, the *L. archibaldi*/Sudan group of parasites was closer to the Ethiopian (0.811<NLi<0.833) and Arabic (0.831<NLi<0.853) strains than to the Kenyan (0.759<NLi<0.861) or Indian (0.785<NLi<0.805) ones (Table S3).

### Cloning and sequence analysis of a selection of 22 RAPD products identified 16 nuclear and 3 minicircle markers

Twenty–two differentially amplified fragments were cloned, sequenced and analyzed *in silico*. These 22 inserts corresponded to 19 different DNA sequences, due to redundancy among the fragments cloned (same size fragments cloned from different DNAs having similar profiles). BLAST analysis allowed identification of significant matching hits (coverage percent>90%; homology percent>80%; e– values <10^−6^) on the *L. infantum* and *L. donovani* reference genomes in 16 cases, which allowed assigning them to 11 chromosomes and mapping them without ambiguities in similar locations in both species (Table 2). Ten of the 11 chromosomes had a large or medium size. 15 markers out of these 16 matched one genomic hit; in case of the marker L1005/320/OPAY8, 3 hits were obtained on *L. infantum* and only one on *L. donovani*. In addition, the chromosomes 36, 30 and 20 had each 2 different markers (6 in total), while 3 markers were identified on chromosome 32. With the exception of one sequence (M106/950/OPAD17), these markers were also conserved in *L. major* with homology ranging from 83% to 100% and coverage from 84% to 99%.

**Figure 3:**
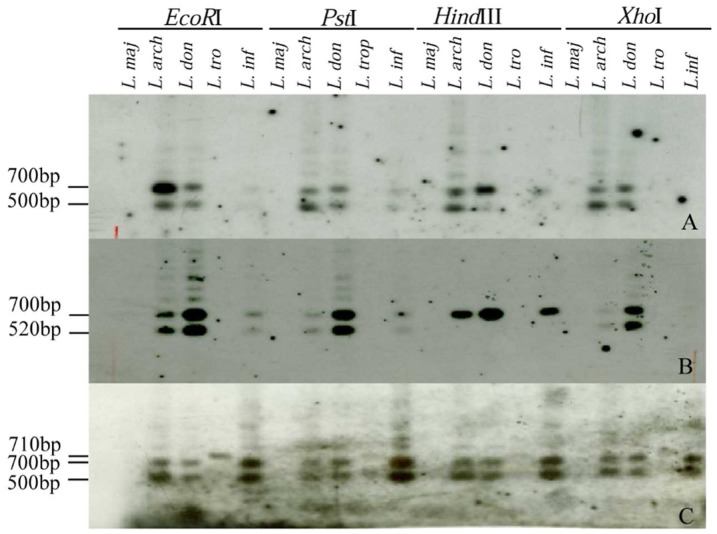
Genomic RFLP analysis using different restriction enzymes and 3 RAPD markers unassigned by *in silico* analysis. The hybridization patterns were revealed by the different probes, A: LEM536/320/OPAY8, B: LEM496/300/OPAD17 and C: M106/200/OPAY9. Tested isolates correspond to *L. major* (FMH; *L. maj*), *L. archibaldi* (GEBRE; *L. arch*), *L. donovani* (HU3; *L. don*), *L. tropica* (DBKM; *L. tro*) and *L. infantum* (LEM1163; *L. inf*). *EcoR*I, *Pst*I, *Hind*III, *Xho*I on top of the panel indicate the restriction enzyme used to digest the total *Leishmania* DNAs.

In case of 3 markers isolated from *L. archibaldi* (M106/200/OPAY9) or *L. donovani* (LEM536/320/OPAY8, LEM496/300/OPAD17), no significant hits were obtained and they could not be assigned to any chromosome or contig on any of the *Leishmania* species genomes (Table 2). To confirm the presence of these 3 unmapped and the M106/950/OPAD17 markers in *Leishmania* parasite genomes, we did a Southern Blot analysis. These markers were radiolabelled and used to probe Southern blots of digested genomic DNAs representative of *L. infantum*, *L. tropica*, *L. major* and *L. donovani* species. The RFLP patterns allowed assignment of the 3 unmapped sequences to kinetoplast DNA; typical hybridization patterns were revealed with signals at 700 bp and 500 bp that were very similar for each genomic DNA tested regardless of the restriction enzyme used. The profiles revealed by these 3 probes were also similar among themselves (Figure 3). This conclusion was also confirmed by positive hybridization of each of these probes to Southern transfers of kDNA minicircles amplified by PCR primers AJS83 & DBY [55] (data not shown).

In case of the M106/950/OPAD17 marker, the genomic Southern blot analysis showed hybridization with *L. donovani* and *L. infantum* DNA but not with *L. major* as predicted by the homology and coverage percent (80% and 51%, respectively); it did react with *L. tropica* DNA (data not shown). This confirms the sequence as an *L. donovani/L. infantum*/*L. tropica* specific marker.

### In silico analysis highlighted features of the RAPD markers and confirmed their potential as polymorphic DNA markers

Nucleotide sequence analysis of the 16 cloned nuclear RAPD markers, using Artemis and ACT software allowed their finer mapping on the respective chromosomes. Synteny was conserved across the species; 11 were in non-coding regions, 3 overlapped coding sequences and 2 were located within coding sequences (Table 2).

Alignment and comparison of the 16 nuclear markers with their respective hits on each of the species genome (*L. donovani*, *L. infantum* and *L. major*) using the Mutation checkup script highlighted inter-specific or intra-specific sequence variability with occurrence of transversions, transitions or indels. Pair wise comparison of the genomic hits in *L. donovani* or *L. infantum* versus *L. major* showed a predominance of transitions (60.67% & 60.39%) over transversions (31.24% & 31.33%) and indels (8.09% & 8.29%) (Table S4). However, the mutations observed comparing *L. infantum* and *L. donovani* hits were largely less abundant than in *L. major* and *L. donovani* or *L. infantum* comparisons with still a predominance of transitions (45.9%). However in contrast, these genomic sites in *L. infantum* and *L. donovani* had similar amounts of transversions (24.59%) and indels (29.51%) (Table S4).

Analysis with Misa cluster conducted on the 16 nuclear markers searched for microsatellites and mapped them on the sequence alignments of the markers and their corresponding genomic sites in *L. infantum* and *L. donovani*. Ten were found to contain at least one microsatellite repeat with two markers having 2 repeats, one having 4 repeats and another 5 (Table 3). So in total, 19 microsatellites and 2 imperfect ones were identified. Motifs repeated contained 1, 2, 3, 4 or 5 nucleotides and belonged to 10 different sequence families (A/T; C; (CA/GT, AC/TG); CT; TA; GGA; GAG; CTTG; CTCC; CACCC); complex motifs associating 2 sequence families were observed in 2 cases (eg T_9_TG_8_). Interestingly, 12 of the 19 microsatellites and one of the imperfect microsatellites were polymorphic among the 3 sets of compared sequences with differences in the number of repeats in most cases. In one case a mutation within the motif created a complex microsatellite in *L. donovani*.

**Table 3:** Selected features characterizing the cloned RAPD markers.

	Chromosomal assignment	Microsatellites polymorphism in the genomic sites	Mismatch in the priming sites	
Markers		Markers	*L. donovani*	*L. infantum*	*L. major*	*L. donovani*		*L. infantum*	
						5'–3'	3'–5'	5'–3'	3'–5'
L1005/220/OPAY8^a^	30	(A)_8_	(A)_9_	(A)_10_	(A)_3_C (A)_6_	+	+	+	+
L1005/650/OPAY14^b^	25	−	−	−	(G)_12_	+	+	+	+
		(CA)_3_(CG)_2_ (CA)_5_	(CA)_3_(CG)_2_ (CA)_5_	(CA)_3_(CG)_2_(CA)_5_	(CA)_12_				
		(CTCC)_3_	(CTCC)_3_	(CTCC)_3_	(CTCC)_3_ *				
		(GGA)_4_ **	(GGA)_4_	(GGA)_4_	(GGA)_4_A (GGA) _3_G_11_ *				
L1005/1000/OPU10^a^	5	(C)_7_	(C)_11_	(C)_10_	(d)(C)_10_	+	+	+	+
		(AC)_7_	(AC)_3_GC (AC)_ 5_	(AC)_7_	(AC)_8_				
		(CACCC)_3_	(CACCC)_3_	(CACCC)_3_	(CACCC)_3_				
		(T)_10_ **	(T)_10_ **	(T)_10_ **	(T)_10_				
		(CT)_5_	(CT)_5_	(CT)_6_	(CT)_4_				
		−	−	−	(CCTCTC)_3_(TC)_6_				
M106/950/OPAD17^a^	36	(T)_10_	(T)_10_	(T)_9_	(T)_10_	+	+	+	+
		−	−	−	(CTCTCG)_3_				
		(T)_9_	(T)_10_	(T)_11_	−				
		(TA)_5_	(TA)_9_	(TA)_9_	(TA)_16_				
LEM138/550/OPAY14^a^	35	(T)_10_	(T)_10_	(T)_10_	(T)_12_ ***	+	+	+	+
LEM980/320/OPAY8^a^	36	(A)_22_ ***	(A)_22_ ***	(A)_20_ ***	(AAC)_6_	+	+	+	+
		(AC)_8_	(AC)_8_	(AC)_9_	(AC)_3_				
LEM719/1000/OPU3^a^	23	(GAG) _4_	(GAG)_4_	(GAG)_4_	GAG	+	+	+	+
LV10/500/OPAD1^b^	20	(GT)_6_	(GT)_6_	(GT)_6_	(GT)_3_ *TT (TG)_7_	+	+	+	+
		(TG)_5_	(TG)_6_	(TG)_6_	−				
LV10/750/OPAY14^a^	17	(CA)_8_	(CA)_3_	(CA)_7_	(CA)_4_ *	+	+	−	+
		(CTTG)_3_	−	−	−				
D14/800/OPE2^c^	32	(T)_3_(TG)_9_	(T)_9_(TG)_8_	(T)_5_(TG)_6_	(T)_6_(TG)_6_ C (TG)_7_ *	+	+	+	+
D14/800/OPAD17^a^	32	−	−	−	(GGGA)_3_	+	+	+	+
LEM138/400/OPAY5^a^	20	−	−	−	−	+	+	+	+
L1005/320/OPAY8^a^	30	−	−	−	−	+	+	+	+
D14/800/OPU10^c^	16	−	−	−	−	+	+	+	+
LV10/700/OPU10^a^	32	−	−	−	−	+	+	+	+
D14/1300/OPAY8^c^	29	−	−	−	−	+	+	+	+
M106/200/OAPY9^d^	kDNA	−	−	−	−	NA	NA	NA	NA
LEM536/320/OPAY8^d^	kDNA	−	−	−	−	NA	NA	NA	NA
LEM496/300/OPAD17^d^	kDNA	−	−	−	−	NA	NA	NA	NA

a : Non coding Sequence;

b : Overlap with a coding sequence;

c : Matching with a coding sequence;

d : Minicircle sequence;

* Imperfect Microsatellite: one mutation in one repeat;

**Imperfect Microsatellite: one mutation in two repeats;

*** Imperfect Microsatellite: one mutation in three repeats; (d): a 58 bp deletion associated to the microsatellite; NA: not applicable; −: no microsatellite observed or no mutations at priming site; +: presence of mismatch at priming site.

Comparison of the 3 sets of sequences with the hits on *L. major* showed that out of the 19 microsatellites, 15 were shared between the 3 species. Among them in *L. major*, 5 corresponded to imperfect repeats and in 2 cases the microsatellites were found complex. This species had in addition 3 unique microsatellites with one being an association of two contiguous microsatellites. In one case, the microsatellite in *L. major* corresponded to simple sequence repeats in the viscerotropic strains.

This brings the total number of microsatellites identified through the RAPD markers in the 4 sets of sequences to 13 sequence families (A/T; C/G; (CA/GT, AC/TG), CT; TA; GGA; GAG; AAC; CTTG; CTCC; GGGA; CACCC; CTCTCG). The sequence of the kDNA markers did not show any microsatellite repeat.

### Analysis of the primer annealing sites highlighted potential mechanisms underlying differential RAPD amplification

Mismatches were observed between the borders of the nuclear markers and their genomic hits in all cases. This prompted analysis of the RAPD priming sites. The nucleotide sequences of primer annealing sites were deduced through alignment of the RAPD primers to the corresponding sites of the markers in *L. donovani* and *L. infantum* genomes. For the 16 nuclear markers, amplified by 9 primers, 77 and 78 mismatches were observed in the RAPD priming sites with these 2 genomes, respectively (Table S5). The priming sites comprised 1 to 3 mutations; in 3 occurrences such mismatches were only observed in one of the 2 priming sites (Table S5). The analysis was further extended, mapping and characterizing the mutations in the priming sites on the 5′ or 3′ parts of the primers here defined as the first 4 and the last 6 nucleotides, respectively [56]. Transversions, transitions and indels accounted for 53.25%–55.12%, 29.49%–31.17% and 8.97%–10.39% of all the mutations, respectively (Table 4). Interestingly, in both species, there was a predominance of mismatches at the 5′ ends (66.66%–68.83%) of the primers than at their 3′ ends (33.33%–31.17%) involving mainly transversions (39.74%–41.56%). In order to assess whether this observation corresponds to a feature unique to the *L. donovani* or *L. infantum* species, the same analysis was done on the corresponding genomic hits in *L. major* genome. This allowed to conclude that the mutations identified also follow the same trend observed, although the proportion of mutations in the 5′ ends were lower (55.92%) and those in the 3′ ends higher (44.09%) than those observed with *L. donovani* and *L. infantum* (Table 4).

**Table 4:** Comparative inter-species analysis of mutations within the RAPD priming sites.

	Transitions (A-G, T-C)	Transversions (A-T, A-C, G-T, G-C)	Indels		All
Mutations	Total at 5′end (%)	Total at 3′end (%)	Total (%)	Total at 5′end (%)	Total at 3′end (%)	Total (%)	Total at 5′end (%)	Total at 3′end (%)	Total (%)	Total 5′	Total 3′
**Primer vs ** ***L. infantum***	17.95	11.54	29.49	39.74	15.38	55.12	8.97	6.41	15.38	66.66	33.33
**Primer vs ** ***L. donovani***	16.88	14.29	31.17	41.56	11.69	53.25	10.39	5.19	15.58	68.83	31.17
**Primer vs ** ***L. major***	13.98	22.58	36.56	34.41	16.13	50.54	7.53	5.38	12.91	55.92	44.09

## Discussion

Polymorphic *Leishmania* markers correlated to clinical or eco-epidemiological features of *Leishmania* parasites are needed for better assessment and monitoring of the disease in endemic areas and transmission foci [57]. They are also relevant to disease control and travel medicine. Parasites causing visceral leishmaniasis belong to at least two species *L. donovani* and *L. infantum* for which genetically differentiated populations have been described in correlation to their geographical origins using different kinds of markers such as gp63 surface antigen–coding genes [27], metabolic enzyme genes [58] or neutral microsatellite sequences [28], [59]. In absence of thorough knowledge on genomes, RAPD remains useful for genetic characterization of a range of organisms including *Leishmania* [29]–[35], [60]. Notably, RAPD genotyping constituted a way of screening bacterial clones or strains to proceed in a cost effective way to NGS analysis of the genomes which in return informed on genomic differences underlying discriminating amplification profiles (genotypes) and confirmed interest of this technique [34], [35]. NGS data analysis also requires skilled personnel in bioinformatics and is time consuming [34].

In case of *Leishmania*, it was mainly used for instance for their taxonomic status elucidation [37] or identification [36], [38] or for their differentiation according to ecological features highlighting correlation to geographical origin [40], [41], [42], [61], or according to cycle components diversity [61]. RAPD also constitutes a powerful mean for the identification of PCR targets, notably for the development of selective or polymorphism specific PCR assays [39]. It has been used to investigate epidemiology of leishmaniasis in diverse settings in addition to other characterization methods [62], [63]. This tool also constitutes powerful means to identify PCR targets for selective or specific amplification of *Leishmania* parasites [39], [44]. Genomes of a limited number of *Leishmania* species and strains are so far available [64]–, so there is for instance little information available on parasites encountered in Africa. Therefore, we associated use of RAPD to the available genome resources to select and characterize by bioinformatics potentially polymorphic markers among VL causing parasites in Africa and elsewhere. Polymorphic potential was inferred from selection of differentially amplified bands, comparisons across the available genomes and annotations.

In a first step during this study, we screened 100 decamer primers over 2 independent rounds of amplifications of one or two sudanese DNAs to select 28 primers generating reproducible amplification profiles. Then, we used this set of 28 decamer primers, not reported to our knowledge elsewhere for *Leishmania* parasites, to screen for polymorphic (differentially amplified) RAPD bands using a panel of 12 parasite strains having different geographical origins in Africa and Asia that were obtained from VL, CL or PKDL cases and assigned to the *L. infantum*, *L. archibaldi* or *L. donovani* species according to their zymodeme. Among a total of 155 amplified bands with this novel set of primers, around 61% of them were polymorphic. Although the number of strains in this study is limited, the results observed corroborate previous published observations. The UPGMA trees built using similarity indexes computed from the profiles obtained with all the primers or a subset of them clearly differentiated the strains identified as *L. donovani* from the *L. infantum* ones, in agreement with the classification of these two species as separate taxonomical entities [16], [17]. In particular *L. infantum* strains belonging to the MON–24 and MON–80 zymodemes were on separate branches from the *L. donovani* strains [22]. In case of the *L. archibaldi* parasites used in this work, the 28 primers clustered them with the *L. infantum* strains while using a reduced set of 9 primers (that were used to amplify the cloned fragments), they clustered with the *L. donovani* strains. RAPD profiles were according to the primers either more similar to *L. infantum* or more similar to *L. donovani*. On cladograms of viscerotropic *Leishmania*, zymodemes attributed to the *L. archibaldi* species form a clade that has intermediate position between *L. infantum* and *L. donovani* [22]. Different studies using different types of markers: microsatellites [18], [28]; genes coding for metabolic enzymes [58] or genes encoding for the Glycoprotein 63 [68] have questioned the taxonomic ranking of *L. archibaldi* as a species. It was hypothesized that in Sudan (East Africa) assignment of *Leishmania* strains to the *L. archibaldi* or *L. infantum* species according to isoenzyme data [22] would indeed result from convergent evolution [18]. Our results tend to confirm questioning of the taxonomic status of *L. archibaldi*. In fact, relatedness of these organisms to the *L. infantum* species will need to be confirmed by more extensive studies comparing for instance the genomes of more extended numbers of African parasites including *L. infantum* from North Africa. One explanation could be the relatedness of the *L. infantum* strains under study to other African viscerotropic *Leishmania*, like *L. donovani*. Indeed, studies using multilocus microsatellite typing of 98 *L. infantum* isolates from different New World foci compared to 308 *L. infantum* and 20 *L. donovani* strains from Old World countries, including Tunisia and Algeria, have shown two main *L. infantum* sub-populations, assigned as MON–1 and non–MON–1 [69]. Interestingly, North African non MON-1 *L. infantum* strains clustered with all the *L. donovani* strains used, their genetic relatedness [69].

Our study showed that the non–typed Sudanese strains had similar profiles to the *L. archibaldi* strains from Sudan or Ethiopia with all primers tested. Although taxonomic relationships of this group of parasites with the other strains varied according to the primers (Figure 2 & Figure S1), consistent observations were made as regards the African *L. donovani* strains, with the Kenyan strains (H9 & LRC-L57) being more similar to the Indian than to the Ethiopian (ADDIS 164) strains. This later one was found more related to the Arabian strain (JEDDAH-KA). Actually, this is in agreement with other studies that had in common a similar set (all or some of these and other strains from the same geographical origins) of *L. donovani* and *L. archibaldi* (GEBRE1) strains and which targeted the intergenic region of gp63 genes [20], ribosomal DNA internal spacer sequences [21] and multilocus microsatellite typing [18], [28], to investigate intraspecific polymorphisms of the *L. donovani* complex. Indeed, our results obtained on a limited number of strains are congruent to these studies that have concluded to a genetic differentiation of the strains according to their geographical origin; similar genetic relationship among these strains were also observed.

Although use of RAPD has been associated to warnings for cautious genetic interpretations because of misleading heterogeneous content or lack of reproducibility [36], [70], RAPD products constitute markers of choice for a range of applications. In case of *Leishmania*, bands consistently amplified were used to identify species [38] or to develop assays for analyzing intra–taxonomic genetic diversity [43] or for species– specific/selective amplification [39], [44]. Here, in absence of genome data of VL causing parasites having different geographical origins, our main objective was to use RAPD as a screening technique and a geographically diverse set of viscerotropic parasites to target bands that are differentially amplified among these parasites to constitute a pool of markers for the development of simple PCR assays for the characterization of viscerotropic *Leishmania* parasites. A selection of such bands have been extracted from the agarose gels, cloned and sequenced. Two cautions were taken, to avoid issues related to reproducibility, only the primers generating reproducible profiles were selected and only one person did the RAPD reactions, using the same protocol, the same reagents (batch and brand) and the same PCR machine. To minimize cloning artifacts, we only selected inserts that had the size expected from the RAPD gels. A total of 22 clones and 19 different sequences have been obtained which were confirmed to belong to the *Leishmania* parasite genomes either by homology searches or by experimental Southern analysis. Extent of coverage and homology percent against *L. donovani* and *L. infantum* genomes indicated perfect matches with the genomic sites. In only one exception, the cloned fragment (D14/1300/OPAY8) contained 2 perfect repeats of a fragment that matched with the 3′ extremity of the LinJ.29.1110 gene. This likely corresponds to an artefactual event that was possibly generated during the RAPD reaction, as described elsewhere [70], [71]. Interestingly, nuclear and kinetoplastic markers were identified in a proportion (15.79% of kDNA) that is within the range described for these genomes in Kinetoplastids (∼5–20%) [72].

All nuclear markers were precisely mapped on the databases while no homologous sequences to the kinetoplast markers were found. This indicates they likely belong to variable regions of minicircles that are so far not described. In addition all (but one) of the nuclear markers were found mapping on medium to large sized chromosomes. Similar observations were made in previous studies where the analyzed markers were also found located on large chromosomes [24], [43]. Four large chromosomes were found to contain 7 markers in total. One explanation could be the polyploidy associated to some chromosomes or the occurrence of amplified DNA arrays [66] that would result in increasing the chances of the primers to anneal to them. Other ones could be linked to the complex molecular mechanisms during RAPD amplification [71] or to a biased distribution of the priming sites. It was previously hypothesized that differences at the annealing sites are responsible for the species–specific amplification of DNA fragments in *Leishmania* that are otherwise conserved across different *Leishmania* species [39]. Here, all the cloned nuclear DNA fragments were shown to match sequences that had polymorphic priming sites. In addition, it was observed that mutations were more important at the 5′ end than at the 3′ end of these sites, which is in agreement with previous studies [56]. These authors proposed that a minimum of 8 nucleotides in a decamer is needed to ensure amplification while perfect matches at the 3′end are not necessary [56]. Another study confirmed these observations and identified in addition mutations at the 3′ end of the priming site as a cause for differential RAPD amplification [35]. In our case, the mutations' number ranged from 0 to 8, and in 14 markers out of 16 we have found that both extremities were polymorphic (Table S5). This strengthens the idea that the primers target polymorphic annealing sites resulting in differential amplification of the bands. Mutations at the priming sites were found to correspond predominantly to transversions. However, the mutations within the RAPD amplified genomic sites corresponded in majority to transitions. All this would indicate that mechanisms underlying differential RAPD amplification among the viscerotropic parasites include primer annealing site variations, and a likely variation in their copy number and/or in their distribution. Of the 16 nuclear markers analyzed, 5 were located either within (2) or overlapped (3) a coding sequence. The remaining ones were in non–coding regions. Interestingly, 10 markers were found to contain single or associated microsatellite repeats. In total, 10 families were identified in the marker or their genomic hits in *L. infantum* and *L. donovani* genomes. Comparison of these sequences further highlighted occurrence of modest expansion of the repeated motives, mutations in the motives that generated imperfect microsatellites or complex association of repeated motives. This supports hypothesis of a preference for the amplification of non–coding polymorphic DNA sequences in relation to clustered organization of priming sites and repetitive sequences [39]. It also informs on evolutionary divergence between *L. major* and *L. donovani*/*L. infantum* in one hand, and between *L. infantum* and *L. donovani* on the other. Closer relationship is highlighted between these two species than with *L. major*, with a trend in having less transitions and transversions and more indels in the RAPD fragments than observed between these species and *L. major*. Observation of microsatellites that are unique to *L. major* or to *L. donovani/L. infantum* is in agreement with previous studies [59]. All this allows predicting potential of these differentially amplified polymorphic markers as useful tools for characterization of viscerotropic parasites. To confirm this hypothesis, we targeted some of these markers to develop in a following study simple assays for differentiation of viscerotropic parasites according to their taxonomy or geographical origin.

## Conclusion

This study associated use of the RAPD technique to public *Leishmania* genome resources to select, clone and characterize *in silico* DNA markers that are differentially amplified among a panel of geographically diverse viscerotropic *Leishmania*. RAPD analysis on this limited panel highlighted polymorphic profiles and was congruent with previous studies emphasizing on close relationships of the parasites, and their clustering according to geographical origin, with parasites from Ethiopia and Sudan being closer between themselves than with parasites from Kenya or India. This work delivers a pool of 19 characterized polymorphic DNA markers that were differentially amplified among these diverse parasites. Mutations at the priming sites and differential representation within these geographically diverse strains seem to underlie their amplification. These markers are also associated to inter– and intra– taxonomic complex sequence and microsatellites variations. They will constitute a pool of targets for development of PCR based assays and could be used for the identification and characterization of viscerotropic *Leishmania* parasites.

## Supporting Information

Figure S1 **UPGMA dendrogram obtained using Nei and Li similarity indexes of the panel of geographically diverse strains using the RAPD profiles generated with the 28 RAPD primers selected for this study.**
(DOCX)

Table S1 **Nucleotide sequences of the 28 RAPD primers used in the present study.**
(DOCX)

Table S2 **Similarity index (Nei and Li, 1979) of the parasites using the RAPD profiles obtained with the 28 RAPD primers selected.**
(DOCX)

Table S3 **Similarity index (Nei and Li, 1979) of the parasites using the RAPD profiles obtained with a selection of 9 RAPD primers used to amplify the cloned RAPD markers.**
(DOCX)

Table S4 **Comparative inter–species analysis of mutations within the genomic hits.**
(DOCX)

Table S5 **Comparative inter–species analysis of mutations within the priming sites.**
(DOCX)
