# Supplementary figures and images for: Screening and Characterization of RAPD Markers in Viscerotropic Leishmania Parasites
Source: PLoS One. 2014 Oct 14;9(10):e109773. doi: 10.1371/journal.pone.0109773 (PMC4196940; doi:10.1371/journal.pone.0109773)

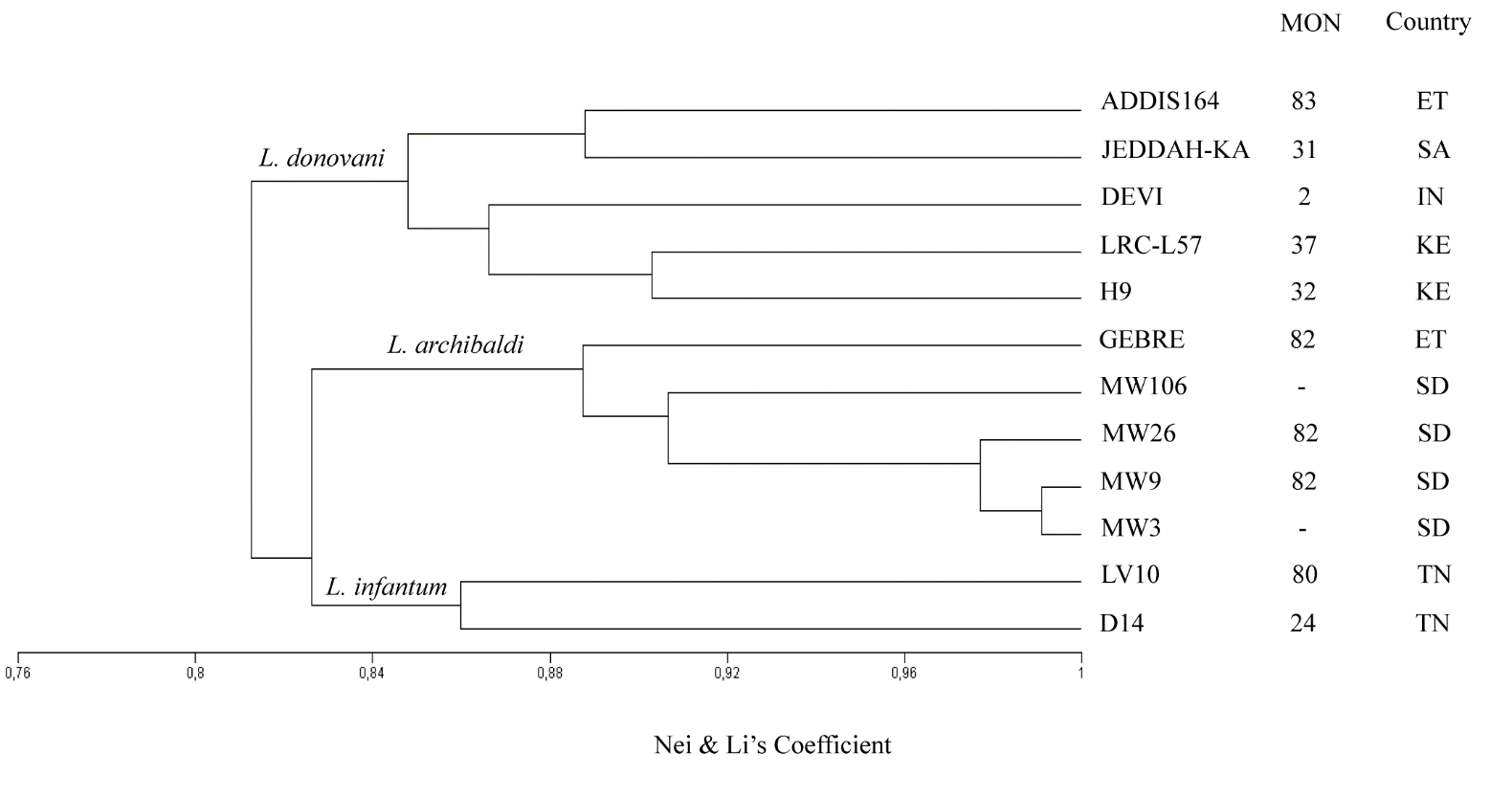

Supplement: Figure S1 — UPGMA dendrogram obtained using Nei and Li similarity indexes of the panel of geographically diverse strains using the RAPD profiles generated with the 28 RAPD primers selected for this study. (DOCX) [file pone.0109773.s001.docx]
