# Supplementary material for: Screening and Characterization of RAPD Markers in Viscerotropic Leishmania Parasites
Source: PLoS One. 2014 Oct 14;9(10):e109773. doi: 10.1371/journal.pone.0109773 (PMC4196940; doi:10.1371/journal.pone.0109773)
Supplement: Table S1 — Nucleotide sequences of the 28 RAPD primers used in the present study. (DOCX) [file pone.0109773.s002.docx]

**Table S1**: Nucleotide sequences of the 28 RAPD primers used in the present study.

| **Primers** | **Nucleotide sequence 5'-3'** |
| --- | --- |
| OP-AD1* | CAAAGGGCGG |
| OP-AD17* | GGCAAACCCT |
| OP-AY5* | TCGCTGCGTT |
| OP-AY8* | AGGCTTCCCT |
| OP-AY9* | CCGATCCAAC |
| OP-AY14* | GGTGGGTAGA |
| OP-U2 | CTGAGGTCTC |
| OP-U3* | CTATGCCGAC |
| OP-U7 | CCTGCTCATC |
| OP-U10* | ACCTCGGCAC |
| OP-U11 | AGACCCAGAG |
| OP-U13 | GGCTGGTTCC |
| OP-U15 | ACGGGCCAGT |
| OP-U17 | ACCTGGGGAG |
| OP-U19 | GTCAGTGCGG |
| OP-O2 | ACGTAGCGTC |
| OP-O4 | AAGTCCGCTC |
| OP-O6 | CCACGGGAAG |
| OP-O7 | CAGCACTGAC |
| OP-O13 | GTCAGAGTCC |
| OP-O16 | TCGGCGGTTC |
| OP-O19 | GGTGCACGTT |
| OP-O20 | ACACACGCTG |
| OP-E2* | GGTGCGGGAA |
| OP-E4 | GTGACATGCC |
| OP-E6 | AAGACCCCTC |
| OP-E7 | AGATGCAGCC |
| OP-E15 | ACGCACAACC |

* RAPD primers that generated the different cloned RAPD markers of this study.
