# Supplementary material for: Screening and Characterization of RAPD Markers in Viscerotropic Leishmania Parasites
Source: PLoS One. 2014 Oct 14;9(10):e109773. doi: 10.1371/journal.pone.0109773 (PMC4196940; doi:10.1371/journal.pone.0109773)
Supplement: Table S2 — Similarity index (Nei and Li, 1979) of the parasites using the RAPD profiles obtained with the 28 RAPD primers selected. (DOCX) [file pone.0109773.s003.docx]

**Table S2**: Similarity index (Nei and Li, 1979) of the parasites using the RAPD profiles obtained with the 28 RAPD primers selected.

| **Strains** | MW106  (SD) | MW26  (SD) | MW9  (SD) | MW3  (SD) | GEBRE1  (ET) | LV10  (TN) | D14  (TN) | JEDDAH-KA  (SA) | H9  (KE) | DEVI  (IN) | LRC-L57  (KE) | ADDIS164  (ET) | | |
| --- | --- | --- | --- | --- | --- | --- | --- | --- | --- | --- | --- | --- | --- | --- |
| MW106 | 1 |  |  |  |  |  |  |  |  |  |  | |  | |
| MW26 | 0,912 | 1 |  |  |  |  |  |  |  |  |  | |  | |
| MW9 | 0,9 | 0,977 | 1 |  |  |  |  |  |  |  |  | |  | |
| MW3 | 0,908 | 0,977 | 0,991 | 1 |  |  |  |  |  |  |  | |  | |
| GEBRE1 | 0,873 | 0,897 | 0,886 | 0,894 | 1 |  |  |  |  |  |  | |  | |
| LV10 | 0,812 | 0,83 | 0,839 | 0,837 | 0,811 | 1 |  |  |  |  |  | |  | |
| D14 | 0,814 | 0,833 | 0,832 | 0,83 | 0,823 | 0,86 | 1 |  |  |  |  | |  | |
| JEDDAH-KA | 0,829 | 0,856 | 0,845 | 0,853 | 0,884 | 0,779 | 0,79 | 1 |  |  |  | |  | |
| H9 | 0,82 | 0,8 | 0,791 | 0,798 | 0,809 | 0,798 | 0,819 | 0,833 | 1 |  |  | |  | |
| DEVI | 0,788 | 0,808 | 0,798 | 0,806 | 0,798 | 0,815 | 0,788 | 0,841 | 0,86 | 1 |  | |  |  |
| LRC-L57 | 0,821 | 0,82 | 0,81 | 0,817 | 0,829 | 0,808 | 0,81 | 0,835 | 0,903 | 0,873 | 1 | |  |  |
| ADDIS164 | 0,804 | 0,824 | 0,813 | 0,821 | 0,843 | 0,772 | 0,774 | 0,888 | 0,839 | 0,837 | 0,903 | | 1 |  |
| SD: Sudan; TN: Tunisia; ET: Ethiopia; SA: Saudi Arabia; KE: Kenya; IN: India | | | | | | | | | | | | | |  |
