# Supplementary material for: Screening and Characterization of RAPD Markers in Viscerotropic Leishmania Parasites
Source: PLoS One. 2014 Oct 14;9(10):e109773. doi: 10.1371/journal.pone.0109773 (PMC4196940; doi:10.1371/journal.pone.0109773)
Supplement: Table S3 — Similarity index (Nei and Li, 1979) of the parasites using the RAPD profiles obtained with a selection of 9 RAPD primers used to amplify the cloned RAPD markers. (DOCX) [file pone.0109773.s004.docx]

**Table S3**: Similarity index (Nei and Li, 1979) of the parasites using the RAPD profiles obtained with a selection of 9 RAPD primers used to amplify the cloned RAPD markers.

| **Strains** | MW106  (SD) | MW26  (SD) | MW9  (SD) | MW3  (SD) | GEBRE1  (ET) | LV10  (TN) | D14  (TN) | JEDDAH-KA  (SA) | H9  (KE) | DEVI  (IN) | LRC-L57  (KE) | ADDIS164  (ET) | |
| --- | --- | --- | --- | --- | --- | --- | --- | --- | --- | --- | --- | --- | --- |
| MW106 | 1,000 |  |  |  |  |  |  |  |  |  |  |  | |
| MW26 | 1 | 1,000 |  |  |  |  |  |  |  |  |  |  | |
| MW9 | 0,974 | 0,974 | 1,000 |  |  |  |  |  |  |  |  |  | |
| MW3 | 0,974 | 0,974 | 1,000 | 1,000 |  |  |  |  |  |  |  |  | |
| GEBRE1 | 0.877 | 0.877 | 0.853 | 0.853 | 1,000 |  |  |  |  |  |  |  | |
| LV10 | 0.757 | 0.757 | 0.763 | 0.763 | 0.712 | 1,000 |  |  |  |  |  |  | |
| D14 | 0.747 | 0.747 | 0.753 | 0.753 | 0.730 | 0.853 | 1,000 |  |  |  |  |  | |
| JEDDAH-KA | 0.853 | 0.853 | 0.831 | 0.831 | 0.838 | 0.693 | 0.684 | 1,000 |  |  |  |  | |
| H9 | 0.779 | 0.779 | 0.759 | 0.759 | 0.763 | 0.753 | 0.769 | 0.769 | 1,000 |  |  |  | |
| DEVI | 0.805 | 0.805 | 0.785 | 0.785 | 0.763 | 0.753 | 0.744 | 0.795 | 0.850 | 1,000 |  |  | |
| LRC-L57 | 0.861 | 0.861 | 0.838 | 0.838 | 0.817 | 0.778 | 0.822 | 0.767 | 0.880 | 0.853 | 1,000 |  | |
| ADDIS164 | 0.833 | 0.833 | 0.811 | 0.811 | 0.845 | 0.694 | 0.685 | 0.849 | 0.747 | 0.773 | 0.857 | 1,000 | |
| SD: Sudan; TN: Tunisia; ET: Ethiopia; SA: Saudi Arabia; KE: Kenya; IN: India | | | | | | | | | | | | |  |
