# Supplementary material for: Screening and Characterization of RAPD Markers in Viscerotropic Leishmania Parasites
Source: PLoS One. 2014 Oct 14;9(10):e109773. doi: 10.1371/journal.pone.0109773 (PMC4196940; doi:10.1371/journal.pone.0109773)
Supplement: Table S4 — Comparative inter–species analysis of mutations within the genomic hits. (DOCX) [file pone.0109773.s005.docx]

|  | | | | | | | | | | | | | | | | | | | | | | | | | | | | | | | | | | | | |
| --- | --- | --- | --- | --- | --- | --- | --- | --- | --- | --- | --- | --- | --- | --- | --- | --- | --- | --- | --- | --- | --- | --- | --- | --- | --- | --- | --- | --- | --- | --- | --- | --- | --- | --- | --- | --- |
| **Table S4:** Comparative inter-species analysis of mutations within the genomic hits. | | | | | | | | | | | | | | | | | | | | | | | | | | | | | | | | | | |  |  |
| **Species** | **Markers** | ***L. donovani* vs *L. infantum*** | | | | | | | | | ***L. donovani* vs *L. major*** | | | | | | | | | | ***L. infantum* vs *L. major*** | | | | | | | | | | | | | |  |  |
|  |  | **Transition** | | **Transversion** | | | | | **Indels** | | **Transition** | | **Transversion** | | | | | **Indels** | | | **Transition** | | | | **Transversion** | | | | | | | **Indels** | | | |  |
|  |  | A-G | T-C | A-T | | A-C | G-T | G-C | 1 or 2 | ≥3 | A-G | T-C | A-T | | A-C | G-T | G-C | 1 or 2 | | ≥3 | A-G | | | T-C | A-T | A-C | | G-T | | G-C | | 1 or 2 | | ≥3 | |  |
| ***L .archibaldi*** | L1005/220/OPAY8 | 1 | 2 | 0 | | 0 | 0 | 0 | 1 | 0 | 8 | 3 | 1 | | 1 | 0 | 1 | 0 | | 0 | 8 | | | 4 | 1 | 1 | | 0 | | 1 | | 0 | | 0 | |  |
|  | L1005/320/OPAY8 | 0 | 0 | 0 | | 0 | 0 | 0 | 0 | 0 | 2 | 10 | 0 | | 0 | 3 | 4 | 1 | | 0 | 2 | | | 10 | 0 | 0 | | 3 | | 4 | | 1 | | 0 | |  |
|  | L1005/650/OPAY14 | 0 | 1 | 0 | | 1 | 1 | 1 | 0 | 0 | 12 | 6 | 1 | | 5 | 4 | 4 | 1 | | 2 | 12 | | | 7 | 1 | 6 | | 5 | | 3 | | 1 | | 2 | |  |
|  | L1005/1000/OPU10 | 3 | 3 | 0 | | 1 | 0 | 0 | 1 | 2 | 30 | 17 | 2 | | 7 | 10 | 7 | 5 | | 5 | 30 | | | 20 | 0 | 8 | | 10 | | 6 | | 7 | | 5 | |  |
|  | M106/950/OPAD17 | 1 | 1 | 0 | | 0 | 0 | 0 | 3 | 0 | 29 | 38 | 9 | | 11 | 11 | 8 | 4 | | 9 | 28 | | | 37 | 9 | 11 | | 11 | | 8 | | 10 | | 3 | |  |
| ***L .donovani*** | LEM138/400/OPAY5 | 0 | 0 | 0 | | 0 | 0 | 1 | 0 | 0 | 12 | 7 | 1 | | 3 | 6 | 2 | 4 | | 1 | 12 | | | 7 | 1 | 3 | | 6 | | 3 | | 4 | | 1 | |  |
|  | LEM980/320/OPAY8 | 1 | 0 | 0 | | 0 | 0 | 0 | 2 | 0 | 10 | 7 | 1 | | 7 | 2 | 3 | 2 | | 2 | 11 | | | 7 | 1 | 7 | | 2 | | 3 | | 2 | | 2 | |  |
|  | LEM138/550/OPAY14 | 0 | 0 | 0 | | 0 | 0 | 0 | 0 | 2 | 18 | 19 | 2 | | 2 | 5 | 4 | 1 | | 7 | 12 | | | 17 | 2 | 2 | | 3 | | 4 | | 1 | | 7 | |  |
|  | LEM719/1000/OPU3 | 2 | 0 | 0 | | 2 | 1 | 0 | 1 | 0 | 45 | 30 | 2 | | 14 | 8 | 8 | 2 | | 4 | 45 | | | 30 | 2 | 14 | | 9 | | 8 | | 2 | | 4 | |  |
| ***L. infantum*** | LV10/500/OPAD1 | 1 | 0 | 0 | | 0 | 0 | 0 | 1 | 0 | 16 | 12 | 0 | | 0 | 5 | 2 | 1 | | 4 | 15 | | | 12 | 0 | 0 | | 5 | | 2 | | 1 | | 4 | |  |
|  | D14/800/OPAD17 | 0 | 2 | 0 | | 0 | 0 | 0 | 1 | 0 | 19 | 34 | 1 | | 4 | 4 | 7 | 3 | | 1 | 19 | | | 32 | 1 | 4 | | 4 | | 7 | | 2 | | 1 | |  |
|  | D14/800/OPE2 | 1 | 1 | 1 | | 0 | 2 | 0 | 1 | 0 | 21 | 19 | 5 | | 12 | 8 | 9 | 2 | | 2 | 21 | | | 18 | 4 | 11 | | 8 | | 10 | | 2 | | 2 | |  |
|  | D14/800/OPU10 | 2 | 2 | 0 | | 0 | 0 | 0 | 0 | 0 | 9 | 6 | 1 | | 0 | 2 | 2 | 0 | | 0 | 11 | | | 4 | 1 | 0 | | 2 | | 2 | | 0 | | 0 | |  |
|  | D14/1300/OPAY8 | 0 | 0 | 0 | | 1 | 0 | 0 | 0 | 0 | 8 | 16 | 2 | | 2 | 2 | 7 | 1 | | 0 | 8 | | | 16 | 2 | 1 | | 2 | | 7 | | 1 | | 0 | |  |
|  | LV10/700/OPU10 | 0 | 1 | 1 | | 0 | 0 | 0 | 0 | 0 | 14 | 17 | 4 | | 2 | 7 | 5 | 4 | | 2 | 14 | | | 16 | 3 | 2 | | 7 | | 5 | | 4 | | 2 | |  |
|  | LV10/750/OPAY14 | 3 | 0 | 1 | | 1 | 0 | 0 | 3 | 0 | 21 | 25 | 4 | | 10 | 6 | 6 | 1 | | 1 | 22 | | | 25 | 5 | 11 | | 6 | | 6 | | 1 | | 1 | |  |
| **Total** | | 15 | 13 | 3 | | 6 | 4 | 2 | 14 | 4 | 274 | 266 | 36 | | 80 | 83 | 79 | 32 | | 40 | 270 | | 262 | | 33 | 81 | | 83 | | 79 | | 39 | | 34 | |  |
| **Total (Transition, Transversion, Indels)** | | 61 | | | | | | | | | 890 | | | | | | | | | | 881 | | | | | | | | | | | | | |  |  |
| **Percentage (%)** | | 24,59 | 21,31 | 4,92 | 9,84 | | 6,56 | 3,28 | 22,95 | 6,56 | 30,79 | 29,89 | | 4,04 | 8,99 | 9,33 | 8,88 | 3,60 | 4,49 | | 30,65 | 29,74 | | | 3,75 | | 9,19 | | 9,42 | | 8,97 | | 4,43 | 3,86 | |  |
| **Global Percentages (%)** | | 45,90 | | 24,59 | | | | | 29,51 | | 60,67 | | | 31,24 | | | | 8,09 | | | 60,39 | | | | 31,33 | | | | | | | | 8,29 | | |  |
