# Supplementary material for: Screening and Characterization of RAPD Markers in Viscerotropic Leishmania Parasites
Source: PLoS One. 2014 Oct 14;9(10):e109773. doi: 10.1371/journal.pone.0109773 (PMC4196940; doi:10.1371/journal.pone.0109773)
Supplement: Table S5 — Comparative inter–species analysis of mutations within the priming sites. (DOCX) [file pone.0109773.s006.docx]

**Table S5** : Comparative inter-species analysis of mutations within the priming sites.

| **Primers** | **Markers** | **Primer vs *L. infantum*** | | | | | | | | | | | | | | | | | | | | | | | | | | | |  |  |
| --- | --- | --- | --- | --- | --- | --- | --- | --- | --- | --- | --- | --- | --- | --- | --- | --- | --- | --- | --- | --- | --- | --- | --- | --- | --- | --- | --- | --- | --- | --- | --- |
|  |  | **Transition** | | | | | | | | **Transversion** | | | | | | | | | | | | | | | | **Indels** | | | | **Total** | |
|  |  | A-G | | | | T-C | | | | A-T | | | | A-C | | | | G-T | | | | G-C | | | | valeur | | | |  | |
|  |  | F | | R | | F | | R | | F | | R | | F | | R | | F | | R | | F | | R | | F | | R | | F | R |
|  |  | 5' | 3' | 5' | 3' | 5' | 3' | 5' | 3' | 5' | 3' | 5' | 3' | 5' | 3' | 5' | 3' | 5' | 3' | 5' | 3' | 5' | 3' | 5' | 3' | 5' | 3' | 5' | 3' |  |  |
| **OPAY5** | LEM138/400 |  |  |  |  |  |  |  |  |  |  |  |  | 1 |  | 1 |  |  |  | 1 |  |  |  |  |  |  |  |  |  | 1 | 2 |
| **OPAY8** | L1005/220 |  |  |  |  |  | 1 |  |  | 1 |  |  |  |  |  |  |  |  |  | 1 |  |  |  |  |  |  |  |  |  | 2 | 1 |
|  | L1005/320 |  |  |  |  |  |  |  | 1 |  |  |  |  | 1 |  | 1 |  | 1 | 1 |  |  |  |  |  |  |  | 2 |  |  | 5 | 2 |
|  | D14/1300 |  |  |  |  |  |  |  |  | 1 |  |  | 1 |  |  |  |  |  |  |  |  |  |  |  |  |  |  | 1 | 2 | 1 | 4 |
|  | LEM980/320 | 1 |  |  |  |  |  |  |  |  |  | 1 |  | 1 |  |  |  |  |  |  |  |  |  | 1 |  |  |  |  |  | 2 | 2 |
| **OPAY14** | L1005/650 |  |  |  | 3 |  |  |  |  |  |  | 1 |  |  | 1 |  |  |  | 1 |  | 1 |  | 1 |  |  |  |  |  |  | 3 | 5 |
|  | LEM138/550 | 1 |  | 1 |  |  |  |  |  |  |  |  |  |  |  |  |  | 1 |  |  |  |  |  |  |  |  | 1 |  |  | 3 | 1 |
|  | LV10/750 |  |  | 1 |  |  |  | 1 |  |  |  |  |  |  |  |  |  |  |  |  |  |  |  | 1 |  |  |  |  |  | **-** | 3 |
| **OPU10** | L1005/1000 | 1 |  | 1 |  |  |  |  |  |  |  | 1 |  | 1 |  |  |  |  |  |  |  |  |  |  |  |  |  |  |  | 2 | 2 |
|  | D14/800 |  |  |  |  |  |  |  |  |  |  |  |  | 1 |  |  |  | 2 |  |  |  |  |  |  |  |  |  | 1 |  | 3 | 1 |
|  | LV10/700 |  |  | 1 |  | 1 |  | 1 |  |  |  |  |  | 1 |  | 1 | 1 |  |  |  |  |  |  |  | 1 |  |  |  |  | 2 | 5 |
| **OPAD17** | D14/800 |  |  |  | 1 |  | 1 |  |  |  | 1 |  | 1 |  |  |  |  |  |  |  |  |  |  | 1 | 1 | 1 |  | 2 |  | 3 | 6 |
|  | M106/950 |  |  |  |  |  |  |  |  |  |  |  |  |  | 1 |  |  |  |  | 1 |  | 1 |  |  |  |  |  |  |  | - | 3 |
| **OPAD1** | LV10/500 |  | 1 |  |  |  |  | 1 | 1 |  |  |  |  |  |  |  |  |  |  |  |  |  |  | 1 |  | 2 |  |  |  | 3 | 3 |
| **OPE2** | D14/800 |  |  | 1 |  |  |  | 1 |  | 1 |  |  |  |  |  |  |  |  |  |  |  |  |  |  |  |  |  |  |  | 1 | 2 |
| **OPU3** | LEM719/1000 |  |  | 1 |  |  |  |  |  |  |  | 1 |  |  |  | 1 |  | 1 |  |  |  |  |  | 1 |  |  |  |  |  | 1 | 4 |
| **Total** | | 3 | 1 | 6 | 4 | 1 | 2 | 4 | 2 | 3 | 1 | 4 | 2 | 6 | 2 | 4 | 1 | 5 | 2 | 3 | 1 | 1 | 1 | 5 | 2 | 3 | 3 | 4 | 2 |  |  |
| **Total F/R** | | 34/44 | | | | | | | | | | | | | | | | | | | | | | | | | | | | | |
| **Total** | | 78 | | | | | | | | | | | | | | | | | | | | | | | | | | | | | |
| **Total (%) at 5' end** | | (14/78) 17,95% | | | | | | | | (31/78) 39,74% | | | | | | | | | | | | | | | | (7/78) 8,97% | | | |  |  |
| **Total (%) at 3' end** | | (9/78) 11,54% | | | | | | | | (12/78) 15,38% | | | | | | | | | | | | | | | | (5/78) 6,41% | | | |  |  |
| F: Forward ; R: Reverse  The number of mismatches observed between the primer sequence and the forward and reverse priming sites on the genomic hit are indicated for each marker. The mutations are  counted according to their nature. The primer is split into its 5’ and 3’ ends. | | | | | | | | | | | | | | | | | | | | | | | | | | | | | | | |

**Table S5 (continued 1) :** Comparative inter-species analysis of mutations within the priming sites.

| **Primers** | **Markers** | **Primer vs *L. donovani*** | | | | | | | | | | | | | | | | | | | | | | | | | | | |  |  |
| --- | --- | --- | --- | --- | --- | --- | --- | --- | --- | --- | --- | --- | --- | --- | --- | --- | --- | --- | --- | --- | --- | --- | --- | --- | --- | --- | --- | --- | --- | --- | --- |
|  |  | **Transition** | | | | | | | | **Transversion** | | | | | | | | | | | | | | | | **Indels** | | | | **Total** | |
|  |  | A-G | | | | T-C | | | | A-T | | | | A-C | | | | G-T | | | | G-C | | | |  | | | |  |  |
|  |  | F | | R | | F | | R | | F | | R | | F | | R | | F | | R | | F | | R | | F | | R | | F | R |
|  |  | 5' | 3' | 5' | 3' | 5' | 3' | 5' | 3' | 5' | 3' | 5' | 3' | 5' | 3' | 5' | 3' | 5' | 3' | 5' | 3' | 5' | 3' | 5' | 3' | 5' | 3' | 5' | 3' |  |  |
| **OPAY5** | LEM138/400 |  |  |  |  |  |  |  |  |  |  |  |  | 1 |  | 1 |  |  |  | 1 |  |  |  |  |  |  |  |  |  | 1 | 2 |
| **OPAY8** | L1005/220 |  |  |  |  |  | 1 |  |  | 1 |  |  |  |  |  |  |  |  |  | 1 |  |  |  |  |  |  |  |  |  | 2 | 1 |
|  | L1005/320 |  |  |  |  |  |  |  | 1 |  |  |  |  | 1 |  | 1 |  | 1 | 1 |  |  |  |  |  |  |  | 2 |  |  | 5 | 2 |
|  | D14/1300 |  |  |  |  |  |  |  |  | 1 |  |  | 1 |  |  |  |  |  |  |  |  |  |  |  |  |  |  | 1 | 2 | 1 | 4 |
|  | LEM980/320 | 1 |  |  |  |  |  |  |  |  |  | 1 |  | 1 |  |  |  |  |  |  |  |  |  | 1 |  |  |  |  |  | 2 | 2 |
| **OPAY14** | L1005/650 |  |  |  | 3 |  |  |  |  |  |  |  |  |  |  |  |  |  |  | 1 | 1 | 1 |  |  |  |  |  |  |  | 1 | 5 |
|  | LEM138/550 | 1 |  | 1 |  |  |  |  |  |  |  |  |  |  |  |  |  |  |  |  |  |  |  |  |  | 1 |  |  |  | 2 | 1 |
|  | LV10/750 |  | 1 | 1 |  |  |  | 1 |  |  |  |  |  |  |  |  |  |  |  |  |  |  |  | 1 |  |  |  |  |  | 1 | 3 |
| **OPU10** | L1005/1000 | 1 |  | 1 | 1 |  |  |  |  |  |  | 1 |  | 1 |  |  |  |  |  |  |  |  |  |  |  |  |  |  |  | 2 | 3 |
|  | D14/800 |  |  |  |  |  |  |  |  |  |  |  |  | 1 |  |  |  | 2 |  |  |  |  |  |  |  |  |  | 1 |  | 3 | 1 |
|  | LV10/700 |  |  | 1 |  | 1 |  | 1 |  |  |  |  |  | 1 |  | 1 | 1 |  |  |  |  |  |  |  | 1 |  |  |  |  | 2 | 5 |
| **OPAD17** | D14/800 |  |  |  | 1 |  | 1 |  |  |  | 1 |  | 1 |  |  |  |  |  |  |  |  |  |  | 1 | 1 | 1 |  | 2 |  | 3 | 6 |
|  | M106/950 |  |  |  |  |  |  |  |  |  |  |  |  |  | 1 |  |  |  |  | 1 |  | 1 |  |  |  |  |  |  |  | 2 | 1 |
| **OPAD1** | LV10/500 |  | 1 |  |  |  |  | 1 | 1 |  |  |  |  |  |  |  |  |  |  |  |  |  |  | 1 |  | 2 |  |  |  | 3 | 3 |
| **OPE2** | D14/800 |  |  | 1 |  |  |  |  |  | 1 |  |  |  |  |  |  |  |  |  | 1 |  |  |  |  |  |  |  |  |  | 1 | 2 |
| **OPU3** | LEM719/1000 |  |  | 1 |  |  |  |  |  |  |  | 1 |  |  |  | 1 |  | 1 |  |  |  |  |  | 1 |  |  |  |  |  | 1 | 4 |
| **Total** | | 3 | 2 | 6 | 5 | 1 | 2 | 3 | 2 | 3 | 1 | 3 | 2 | 6 | 1 | 4 | 1 | 4 | 1 | 5 | 1 | 2 | 0 | 5 | 2 | 4 | 2 | 4 | 2 |  |  |
| **Total F/R** | |  | 32/45 | | | | | | | | | | | | | | | | | | | | | | | | | | |  |  |
| **Total** | | 77 | | | | | | | | | | | | | | | | | | | | | | | | | | | |  |  |
| **Total (%) at 5' end** | | (13/77) 16,88% | | | | | | | | (32/77) 41,56% | | | | | | | | | | | | | | | | (8/77) 10,39% | | | |  |  |
| **Total (%) at 3' end** | | (11/77) 14,29% | | | | | | | | (9/77) 11,69% | | | | | | | | | | | | | | | | (4/77) 5,19% | | | |  |  |
| F: Forward ; R: Reverse | | | | | | | | | | | | | | | | | | | | | | | | | | | | | | | |

**Table S5 (continued 2) :** Comparative inter-species analysis of mutations within the priming sites.

| **Primers** | **Markers** | **Primer vs *L. major*** | | | | | | | | | | | | | | | | | | | | | | | | | | | |  |  |
| --- | --- | --- | --- | --- | --- | --- | --- | --- | --- | --- | --- | --- | --- | --- | --- | --- | --- | --- | --- | --- | --- | --- | --- | --- | --- | --- | --- | --- | --- | --- | --- |
|  |  | **Transition** | | | | | | | | **Transversion** | | | | | | | | | | | | | | | | **Indels** | | | | **Total** | |
|  |  | A-G | | | | T-C | | | | A-T | | | | A-C | | | | G-T | | | | G-C | | | |  | | | |  |  |
|  |  | F | | R | | F | | R | | F | | R | | F | | R | | F | | R | | F | | R | | F | | R | | F | R |
|  |  | 5' | 3' | 5' | 3' | 5' | 3' | 5' | 3' | 5' | 3' | 5' | 3' | 5' | 3' | 5' | 3' | 5' | 3' | 5' | 3' | 5' | 3' | 5' | 3' | 5' | 3' | 5' | 3' |  |  |
| **OPAY5** | LEM138/400 |  |  |  |  |  |  |  |  |  |  |  |  | 1 |  | 1 |  |  |  | 1 |  |  |  |  |  |  |  |  |  | 1 | 2 |
| **OPAY8** | L1005/220 |  |  |  | 1 |  | 1 | 1 |  | 1 |  | 1 |  |  |  |  |  |  |  |  |  |  |  |  |  |  |  |  |  | 2 | 3 |
|  | L1005/320 |  |  |  |  |  |  |  | 1 |  |  |  |  |  |  | 1 |  | 1 | 1 |  |  |  |  |  |  |  | 2 |  |  | 4 | 2 |
|  | D14/1300 |  |  |  |  |  |  |  |  | 1 |  |  | 1 |  |  |  |  |  |  |  |  |  |  |  |  |  |  | 1 | 2 | 1 | 4 |
|  | LEM980/320 |  |  |  |  |  |  |  |  |  |  | 1 |  | 1 |  |  |  |  |  |  |  |  |  | 1 |  |  |  |  |  | 1 | 2 |
| **OPAY14** | L1005/650 |  |  |  | 3 |  |  |  |  |  |  |  |  |  | 1 |  |  |  |  |  | 1 | 1 |  | 1 |  |  |  |  |  | 2 | 5 |
|  | LEM138/550 | 1 |  | 1 |  |  |  | 1 |  |  |  |  |  |  |  |  |  | 1 |  |  |  |  |  |  |  |  | 1 |  |  | 3 | 2 |
|  | LV10/750 |  | 1 | 1 |  |  |  | 2 |  |  |  |  | 1 |  |  |  |  |  |  |  |  |  |  | 1 | 1 |  |  |  |  | 1 | 6 |
| **OPU10** | L1005/1000 | 1 | 2 | 1 | 1 |  |  |  |  |  |  | 1 |  | 1 |  |  |  |  |  |  |  |  |  |  |  |  |  |  |  | 4 | 3 |
|  | D14/800 |  |  |  |  |  |  |  |  |  |  |  |  | 1 |  |  |  | 2 |  |  |  |  |  |  |  |  |  | 1 |  | 3 | 1 |
|  | LV10/700 |  |  |  | 1 | 1 |  | 1 |  |  |  |  |  | 1 |  | 1 | 2 |  |  |  |  |  |  |  |  |  |  |  |  | 2 | 5 |
| **OPAD17** | D14/800 |  |  |  | 1 |  | 1 |  | 2 |  | 1 |  | 1 |  |  |  |  |  |  | 1 |  |  |  |  | 1 | 1 |  | 2 |  | 3 | 8 |
|  | M106/950 |  |  |  |  |  |  |  | 1 |  |  |  |  |  | 1 |  |  | 1 |  | 1 |  |  | 1 |  |  |  |  |  |  | 3 | 2 |
| **OPAD1** | LV10/500 |  | 1 |  |  |  |  |  | 3 |  |  | 1 |  |  |  |  | 1 |  |  | 1 |  |  |  |  |  | 2 |  |  |  | 3 | 6 |
| **OPE2** | D14/800 | 1 |  | 1 |  |  |  |  |  | 1 |  | 1 |  |  |  |  |  |  |  |  | 1 |  |  |  |  |  |  |  |  | 2 | 3 |
| **OPU3** | LEM719/1000 |  |  |  | 1 |  |  |  |  |  |  |  |  |  |  | 1 |  | 1 |  |  |  |  |  | 1 |  |  |  |  |  | 1 | 3 |
| **Total** | | 3 | 4 | 4 | 8 | 1 | 2 | 5 | 7 | 3 | 1 | 5 | 3 | 5 | 2 | 4 | 3 | 6 | 1 | 4 | 2 | 1 | 1 | 4 | 2 | 3 | 3 | 4 | 2 |  |  |
| **Total F/R** | | 36/57 | | | | | | | | | | | | | | | | | | | | | | | | | | | |  |  |
| **Total** | | 93 | | | | | | | | | | | | | | | | | | | | | | | | | | | |  |  |
| **Total (%) at 5' end** | | (13/93) 13,98% | | | | | | | | (32/93) 34,41% | | | | | | | | | | | | | | | | (7/93) 7,53% | | | |  |  |
| **Total (%) at 3' end** | | (21/93) 22,58% | | | | | | | | (15/93) 16,13% | | | | | | | | | | | | | | | | (5/93) 5,38% | | | |  |  |
| F: Forward ; R: Reverse | |  | | | | | | | |  | | | | | | | | | | | | | | | |  | | | |  |  |
